# Efficacy and Safety of Vitrectomy without Using Perfluorocarbon Liquids and Drainage Retinotomy Associated with Postoperative Positioning Based on Residual Subretinal Fluid for Rhegmatogenous Retinal Detachment

**DOI:** 10.1155/2021/5588479

**Published:** 2021-04-20

**Authors:** Paolo Chelazzi, Claudia Azzolini, Claudia Bellina, Francesca Cappelli, Ilaria Del Genovese, Laura Caraffa, Francesco Scullica

**Affiliations:** ^1^Department of Ophthalmology, Istituto Clinico Città Studi, Milan, Italy; ^2^Department of Ophthalmology, Multimedica Hospital, Castellanza, Italy

## Abstract

Medical records of 75 eyes from 75 consecutive patients with uncomplicated rhegmatogenous retinal detachment (RRD) who underwent pars plana vitrectomy (PPV) were analyzed. Inclusion criteria were patients with RRD who underwent primary 23- or 25-gauge PPV with air, gas, or SiO tamponade and performed by a single surgeon, no use of perfluorocarbon liquids (PFCL) and drainage retinotomy, and follow-up ≥ six months. Exclusion criteria were patients who underwent previous vitreoretinal surgery, proliferative vitreoretinopathy (PVR) more than grade B, giant tears, and encircling band associated with PPV. The main endpoint was the anatomical retinal reattachment rate after a single surgical procedure. Secondary endpoints were best-corrected visual acuity (BCVA), postoperative retinal displacement, and intraoperative and/or postoperative complications. Primary anatomical success was achieved in 97.3% of cases using this modified surgical procedure. Retinal slippage occurred only in 28.2% of patients and it was not observed in all cases of macula-on RRD. The mean logMAR of the BCVA significantly improved in 92% of patients and no intraoperative complications were observed. The results suggest that complete subretinal liquid drainage is not mandatory for all RRD cases treated with PPV and that using PFCL and performing a drainage retinotomy are not essential in eyes with primary RRD and PVR less than grade B. Postoperative positioning after PPV for uncomplicated RRD based on the presence or absence of residual subretinal fluid at the end of surgery could limit the occurrence of postoperative retinal displacement, while promoting patient compliance.

## 1. Introduction

Rhegmatogenous retinal detachment (RRD) is a sight-threatening condition and it may be considered one of the few ocular emergencies. This potentially blinding pathology is defined as the separation of the neurosensory retina from the underlying retinal pigment epithelium [1].

The prevalence of RRD ranges from 6.3 to 17.9 per 100,000 and the largest incidence of cases is in the population between 60 and 70 years of age, with a secondary peak in young myopes and a higher overall incidence in men [2].

In case of RRD, surgery is the only effective treatment. The choice of surgical technique varies significantly between surgeons, with pars plana vitrectomy (PPV) recently becoming the most used method [3, 4]. PPV has been reported as a successful treatment for RRD in many studies [4–9]. According to SPR Study, this surgical technique in some cases may be preferred over scleral buckling [10]. In the last few years, the improvement of surgical instruments and viewing systems allowed minimally invasive PPV to reach a higher surgical success rate and less surgical complications, in addition to having some advantages such as mild surgical trauma [11, 12].

Chang et al. first introduced perfluorocarbon liquids (PFCL) as a surgical tool for the management of RRD during PPV [13, 14]. Since then and over the past thirty years, heavy fluids became widespread in common use during retinal detachment surgery. Their main purpose is to reattach the detached retina, while subretinal fluid is drained via a peripheral break and to protect the retina during anterior vitrectomy as well as to photocoagulation and cryopexy. Despite its undisputed benefits, especially for the treatment of complex retinal detachments, PFCL showed signs of chemical and mechanical toxicity to ocular tissues [15–17]. Moreover, the risk of intraocular retention of PFCL cannot be completely avoided after its usage [18–20].

It has been recently reported that complete subretinal liquid drainage during PPV is not mandatory for all RRD surgical procedures, showing that the use of PFCL can be avoided in case of uncomplicated macula-off RRD with peripheral retinal breaks [21].

The aim of this study was to evaluate the outcome of primary PPV in the treatment of RRD without the intraoperative use of PFCL and drainage retinotomy.

## 2. Materials and Methods

This is a retrospective study conducted in compliance with the current version of the Declaration of Helsinki and with the Health Insurance Portability and Accountability Act regulations, as well as all national legal and regulatory requirements.

Medical records of 75 eyes from 75 consecutive patients with uncomplicated RRD who underwent PPV were analyzed. Inclusion criteria were (1) patients with RRD who underwent primary 23- or 25-gauge PPV with air, gas (C3F8 12% or SF6 20%), or silicone oil (SiO) tamponade and performed by a single surgeon (PC), (2) PFCL was not used and posterior drainage retinotomy was not performed, and (3) follow-up ≥ six months. Exclusion criteria were (1) patients who underwent previous vitreoretinal surgery, (2) proliferative vitreoretinopathy (PVR) more than grade B, (3) giant tears, and (4) encircling band associated with PPV.

The main endpoint was the evaluation of the anatomical retinal reattachment rate after a single surgical procedure. Primary success rate was defined as the presence of a reattached retina six months after PPV in case of air or gas tamponade or six months after SiO surgical removal in case of SiO tamponade. The secondary endpoints were (1) best-corrected visual acuity (BCVA), (2) postoperative retinal displacement, (3) intraoperative complications such as iatrogenic retinal breaks and lens injury, and (4) other reported complications.

Preoperative data recorded included logarithm of the minimum angle of resolution (logMAR) BCVA tested on the Early Treatment Diabetic Retinopathy Study (ETDRS) chart at the distance of 4 meters, retinal detachment location and quadrants involved, retinal breaks location and number, macula and lens status, and demographics.

Postoperative data collected included PPV surgery platform (25- or 23-gauge), intraoperative retinal tamponade (air, C3F8 12% or SF6 20%, SiO), cataract extraction at the time of PPV, anatomical primary success rate, BCVA, presence of metamorphopsia (detected with Amsler grid), intraocular pressure (IOP measured by Goldmann applanation tonometer), and any others intraoperative or postoperative complications. Patients were routinely followed up at 1, 3, and 6 months after surgical procedures. Autofluorescence imaging (FAF) and spectral-domain optical coherence tomography (SD-OCT) were performed three weeks after surgery in case of air or SF6 tamponade and six weeks after surgery in case of C3F8 tamponade. In case of SiO using, FAF and SD-OCT were obtained one month after its surgical removal.

### 2.1. Imaging

Images were recorded with Spectralis HRA + OCT (Heidelberg Engineering, Germany), which combines a confocal scanning laser ophthalmoscope with spectral-domain OCT (with a barrier filter at 500 nm and an excitation wavelength at 488 nm). Macula status was confirmed by preoperative SD-OCT scans when achievable.

### 2.2. Surgical Technique

All surgeries were performed under local anesthesia obtained by retrobulbar block. A standard core and peripheral 25- or 23-gauge PPV (Stellaris, Bausch & Lomb, New York, USA) was performed in all patients. A noncontact wide-angle viewing system was used for the visualization during PPV for the repair of retinal detachment. A posterior vitreous detachment was induced by enhanced suction with the vitrectomy probe around the optic nerve when necessary. After a complete peripheral PPV under scleral indentation, the vitreous on retinal tears was shaved to relieve traction and to favor fluid exchange. Under any circumstances, PFCL and posterior drainage retinotomy were used. Subsequently, subretinal fluid was drained from the midperipheral or peripheral break (or breaks) through the vitrectomy probe or via an extrusion cannula with a silicone tip (fluid-fluid exchange) as much as possible. After that, fluid/air exchange was performed while maintaining passive drainage through the retinal break (if single) or through the main or more posterior break (if multiple) or from both latter if the visibility allowed it. In case of macula-off RRD, any residual fluid under the macula at the end of the procedure was left. In case of macula-on RRD, the procedure was interrupted just in case of the approach of the subretinal fluid to the macular area and repeated (after refilling the vitreous chamber with balanced salt solution). In the few cases of further persistence of the risk of fluid passage under the macula, the reason was assigned to the presence of multiple retinal tears in the opposite quadrants. Therefore, a twin-light fiber chandelier illumination system [22] was used to allow bimanual double drainage from the opposite breaks both actively with the vitrectomy probe and passively with the extrusion cannula. After that, endolaser or transscleral cryopexy treatments were performed around the retinal break or breaks. Finally, air or gas (C3F8 12% or SF6 20%) was used as retinal tamponade in presence of RRD without PVR or with PVR grade A, whereas SiO was used in the case of RRD with PVR grade B.

If necessary, combined surgery (PPV plus cataract extraction and IOL implantation) was performed.

Patients were placed supine after surgery in case of macula-off RRD (with fluid under the macula at the end of the procedure) or in case of complete intraoperative drainage of the subretinal fluid. If complete drainage of the subretinal fluid was not achievable, patients were placed prone in the operating room immediately after surgery for a few hours and, after that, they were asked to maintain a position alternatively supine or corresponding to the break causes of the RRD.

### 2.3. Statistical Analysis

According to Holladay, counting fingers was assigned 2.3 logMAR, hand movement 2.6 logMAR, and light perception 2.9 logMAR [23]. All continuous data were expressed as mean ± standard deviation (SD). The statistical analysis was carried out using SPSS 26.0 statistical software (SPSS, Inc., Chicago, IL, USA). *P* value < 0.05 was considered statistically significant.

## 3. Results

**Figure 1:**
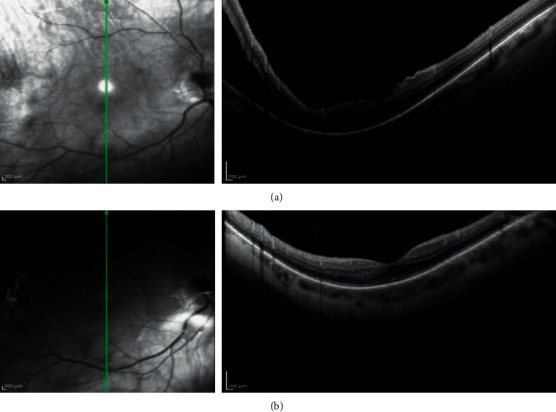
Preoperative SD-OCT image (a) and six weeks' postoperative SD-OCT image (b) obtained after macula-on RRD surgery. Despite the proximity of the subretinal fluid to the macula (a), the anatomical reattachment of the retina without macular changes (b) demonstrates that the modified surgical procedure used in this study to treat uncomplicated RRD is efficacy.

**Figure 2:**
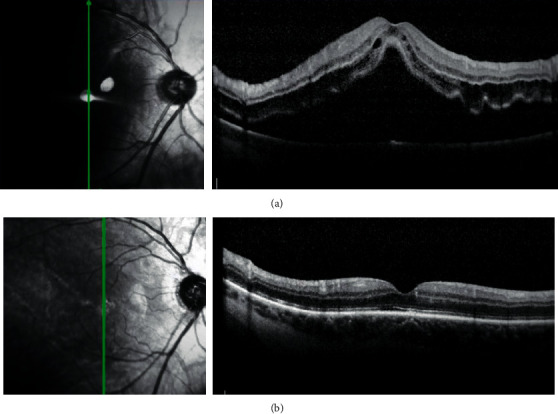
Preoperative SD-OCT image (a) and six weeks' postoperative SD-OCT image (b) obtained after macula-off RRD surgery. Despite the fact that a residual fluid under the macula at the end of the surgical procedure was left, the anatomical macula reattachment appears satisfactory (b).

A total of 75 eyes from 75 consecutive patients (44 men, 31 women) with RRD were included in this study according to the inclusion and exclusion criteria. All of them underwent the previously described surgery at three institutional centers. They were followed up for at least six months. The mean age was 59.1 ± 10.3 years. 58 eyes (77.3%) were pseudophakic and 17 (22.7%) were phakic. Phacoemulsification was performed together with PPV in 9 patients (52.9% of phakic eyes). 34 cases were macula-on (45.3%) and 41 cases were macula-off (54.7%) (Figures 1 and 2). Of the latter, preoperative SD-OCT detected an attached fovea in 5 eyes (6.7%) (Table 1).

RRD was located in the superior hemisphere in 34 eyes (45.3%) and in the inferior hemisphere in 14 eyes (18.7%) and involved both the superior and inferior hemispheres in 27 eyes (36%). Retinal breaks were detected in the upper quadrants in 53 eyes (70.7%), in the lower quadrants in 7 eyes (9.3%), and in both upper and lower quadrants in 15 eyes (20%) (Table 2).

25-gauge PPV was performed in 51 eyes (68%) and 23-gauge PPV in the other 24 eyes (32%). 59 eyes were tamponed with gas (78.7%), 44 of which with C3F8 (58.7%) and 15 with SF6 (20%); 10 eyes were tamponed with air (13.3%) and 6 eyes with 1000 centistokes SiO (8%), which was removed 2.6 ± 0.7 (mean ± SD) months after the first operation (Table 3).

PFCL has never been used and no posterior drainage retinotomy was performed during any surgery. No intraoperative complications were observed.

73 eyes (97.3%) achieved primary anatomical retinal reattachment after a single surgical procedure. 2 eyes required additional surgery because of PVR and were successfully treated with SiO injection. At the end of follow-up, retina reattachment rate was 100%.

The mean logMAR of the BCVA significantly improved from 1.08 ± 0.8 to 0.45 ± 0.3 (*P* < 0.001). BCVA improved in 69 eyes (92%) and BCVA did not decline in any patient.

**Figure 3:**
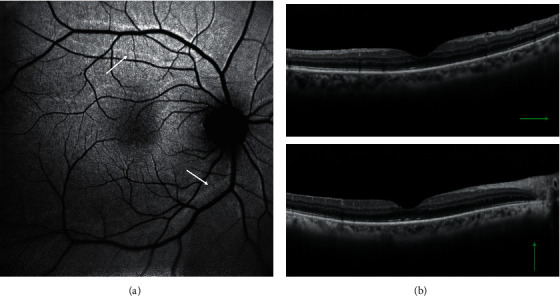
FAF (a) and SD-OCT (b) images six weeks after macula-off RRD surgery. White arrows pointing to hyperfluorescent lines (a) indicate the preoperative position of retinal vessels. Retinal displacement was not observed in cases of macula-on RRD.

Postoperative retinal displacement was evaluated on 71 eyes analyzing FAF images, since four cases were excluded due to the absence of available images. 11 of 39 eyes affected by macula-off RRD left showed a postoperative retinal slippage on FAF imaging (Figure 3). Retinal displacement was not observed in any case of macula-on RRD. A significant association between retinal displacement and postoperative metamorphopsia was found. 8 of 11 patients with retinal shift did report distorted vision (*P* < 0.001). However, the displacement of the retina did not affect the BCVA at the end of the follow-up period.

Other postoperative reported complications included epiretinal membrane formation in 22 eyes (29.3%) and cystoid macular edema in 8 eyes (10.7%); both conditions were detected in OCT scans obtained during the follow-up period. An elevation of the postoperative IOP (>20 mmHg) was observed in 15 eyes (20%), which normalized in all after the start of topical IOP lowering medication (Table 4).

## 4. Discussion

RRD occurs when fluid from the vitreous cavity passes through a retinal break into the subretinal space, causing the separation of the neurosensory retina from the underlying retinal pigment epithelium. Vitreoretinal changes and anatomic variations that predispose to retinal break formation and retinal detachment are represented by vitreous body liquefaction and syneresis leading to tractional forces on the retina and by developmental or acquired degenerative retinal or vitreoretinal abnormalities which will become the site of the break.

The purpose of retinal reattachment surgery is to bring the detached retina into permanent contact with the underlying retinal pigment epithelium, sealing all retinal breaks and relieving vitreous traction [1]. In the past years, different surgical techniques have been proposed to manage RRD, including pneumatic retinopexy [24], scleral buckling [25], PPV [26], and a combination of previous techniques [27]. In recent years, PPV has become the preferred surgical technique in cases of uncomplicated RRD [28].

The increasing popularity of PPV is partly due to the advent of transconjunctival sutureless small gauge technique [29] and partly to the development of surgical adjuvant technologies such as increased vitreous cutter speeds, wide-angle viewing systems, chandelier lights, curved and lighted instruments, and surgical devices such as intraocular gases and PFCL. For all these reasons, the success rates of PPV for RRD have recently increased, reaching up to 100% of the final anatomical success rate in some cases [8]. However, it is not always necessary to use all surgical devices currently available and they can be even harmful in some cases.

The retinal toxicity of PFCL has been documented first *in vitro* studies and in rabbits [30] and later in clinical follow-up [18]. Its related ocular inflammation is observed especially in cases of subretinal PFCL [19]; in these cases, a significant pigment epithelial atrophy and local functional changes in the retinal sensitivity may occur [18]. In cases of persistent inflammation (especially in young patients), secondary membrane formation or recurrent RRD, reoperation for removal of PFCL is necessary [31, 32]. Interaction between PFCL and SiO or heavy SiO is involved in the occurrence of sticky oil formation [33]. In addition, PFCL droplets may move into the anterior chamber causing corneal endothelial damage [34]. Moreover, the usage of PFCL increases surgical maneuvers and the associated risks, extending the operation time and increasing expenses.

Besides to the use of PFCL, when subretinal fluid cannot be completely drained via the original retinal breaks, it can be drained via a small posterior retinotomy site. However, posterior retinotomy may cause proliferation and visual field defects [35].

Recent studies have shown the safety and efficacy of PPV for the repair of uncomplicated RRD without the use of PFCL, with both gas [21, 36] and air [37, 38] tamponade. These studies also confirmed that anatomical retinal reattachment after PPV can be successfully achieved without complete drainage of subretinal fluid in the same way as observed in pneumatic retinopexy [39] and in scleral buckling [40]. The residual subretinal liquid can be spontaneously absorbed by the retinal pigment epithelium during the first postoperative hours, if the retinal breaks are sealed [41].

Even though the number of anatomical surgical successes in retinal reattachment after PPV has increased in recent years, Dell'Omo et al. reported unintentional displacement of the retina after the repair of primary and uncomplicated macula-off RRD with PPV in more than one-third of cases, despite postoperative prone posturing taken immediately after surgery and kept for 24 hours [42]. Other studies showed that the use of intraoperative PFCL seems to be associated with lower occurrence of retinal shift after PPV for macula-off RRD [43, 44]. Furthermore, even though previous studies suggested that the use of early face-down positioning in eyes with RRD treated with PPV and gas tamponade may prevent retinal displacement after surgery [45], it has recently been demonstrated that not only does postoperative posture seem to influence postoperative macular shift after surgery [43, 44, 46], but that this event seems to occur less than in other cases (range, 35%–72%) [42, 45, 47–49]. However, it is not clear whether the low reported rate of retinal shift is due to the no-prone posture or to the use of intraoperative PFCL in these cases. Moreover, the postoperative displacement of the retina can also happen in case of macula-on RRD [47], stressing the importance of avoiding the passage of fluid under the macula during intraoperative maneuvers.

In the current study, all eyes with primary RRD and PVR less than grade B who were eligible for PPV underwent the described surgical technique and were tamponed with air, gas (C3F8 12% or SF6 20%), or SiO depending on the severity of RRD. During fluid/air exchange, great care was taken to fully passively extract fluid under the retina through retinal breaks, especially in the case of macula-on RRD. Not even in case of approach of the subretinal fluid to the macular area, PFCL was used or posterior drainage retinotomy was performed; in such cases fluid/air exchange was repeated or, if necessary, a bimanual double drainage was performed. Postoperative positioning was decided exclusively by the presence or absence of evident residual subretinal fluid at the end of surgery and was not based on the location of retinal breaks.

We reached a satisfactory primary anatomical success (97.3%) by use of these intraoperative and postoperative modified procedures. Postoperative retinal slippage occurred only in 28.2% of patients and it was not observed in all cases of macula-on RRD, showing the effectiveness of the modified fluid/air exchange technique performed. The mean logMAR of the BCVA significantly improved in 92% of patients and no intraoperative complications were observed.

In accordance with previous observations, we found that using PFCL and performing a drainage retinotomy are not always necessary and they should be reserved for more complicated RRD cases [37, 38, 50]. We believe that complete drainage of subretinal fluid is not necessary except for patients who undergo SiO tamponade, to avoid the risk of PVR formation and recurrence of RRD due to incomplete filling of the vitreous chamber by SiO [51]. We also hypothesize that postoperative supine positioning in case of macula-off RRD with fluid under the macula at the end of the procedure could limit the risk of retinal displacement after PPV.

We acknowledge several limitations to our study. Firstly, the study was retrospective and there was no control group. Secondly, the sample analyzed was relatively small. Thirdly, all patients underwent a single surgeon operation and the choice of tamponade was made depending on the severity of RRD judged by the surgeon.

## 5. Conclusions

A modified procedure for the treatment of uncomplicated RRD was assessed. This technique included both a PPV without the use of PFCL and posterior drainage retinotomies and postoperative positioning based on the presence or absence of residual subretinal fluid at the end of surgery. The results of this study suggest that complete subretinal liquid drainage is not mandatory for all RRD cases treated with PPV and that using PFCL and performing a drainage retinotomy are not essential in eyes with primary RRD and PVR less than grade B; they should rather be reserved for more complicated RRD cases. Moreover, postoperative positioning after PPV for uncomplicated RRD based on the presence or absence of residual subretinal fluid at the end of surgery could limit the occurrence of postoperative retinal displacement, while promoting patient compliance. An additional large randomized controlled study on this combined intraoperative and postoperative procedure could demonstrate its indication in case of RRD.

## Figures and Tables

**Table 1:** Baseline characteristics expressed as mean ± SD and percentage (75 patients).

Age (years ± SD)	59.1 ± 10.3
Male (%)	44 (58.7)
Baseline BCVA (logMAR ± SD)	1.08 ± 0.8
RRD macula status (%)	
On	34 (45.3)
Off	41 (54.7)
Fovea attached	5 (6.7)
Lens status (%)	
Pseudophakia	58 (77.3)
Phakia	17 (22.7)

**Table 2:** Locations of RRD and retinal breaks (75 patients).

RRD hemisphere (%)	Superior 34 (45.3)
	Inferior 14 (18.7)
	Superior + inferior 27 (36)

Retinal breaks quadrants (%)	Upper 53 (70.7)
	Lower 7 (9.3)
	Upper + lower 15 (20)

**Table 3:** Surgical procedures (75 patients).

*Gauge*	(%)
23	24 (32)
25	51 (68)

*Tamponade*	(%)
Gas	59 (78.7)
C3F8 20%	44 (58.7)
SF6 12%	15 (20)
Air	10 (13.3)
SiO	6 (8)

**Table 4:** Study endpoints.

*Anatomical retinal reattachment rate (75 patients)*	(%)

Single procedure	73 (97.3)
Additional surgery	2 (2.7)
*BCVA (75 patients)*	(±SD)
Baseline	1.08 ± 0.8
Last follow-up	0.45 ± 0.3^*∗*^
*Retinal displacement (71 patients)*	
Macula-on RRD (32)	0
Macula-off RRD (39)	
Slippage	11
Associated metamorphopsia	8/11^*∗*^
*Complications*	(%)
Epiretinal membrane	22 (29.3)
Cystoid macular edema	8 (10.7)
IOP > 20 mmHg	15 (20)

^*∗*^ *P* value < 0.001.

## Data Availability

The data used to support the findings of this study are available from the corresponding author upon reasonable request.
